# Assessing Recovery from Mild Traumatic Brain Injury (Mtbi) using Magnetoencephalography (MEG): An Application of the Synchronous Neural Interactions (SNI) Test

**DOI:** 10.29245/2572.942x/2020/3.1274

**Published:** 2020-09-03

**Authors:** Don R. Thorpe, Brian E. Engdahl, Arthur Leuthold, Apostolos P. Georgopoulos

**Affiliations:** 1 Graduate Program in Cognitive Science, University of Minnesota, Minneapolis, Minnesota, USA; 2 Brain Sciences Center, Minneapolis VA Medical Center, Minneapolis, Minnesota, USA; 3 Department of Neuroscience, University of Minnesota, Minneapolis, Minnesota, USA; 4 Department of Psychology, University of Minnesota, Minneapolis, Minnesota, USA; 5 Department of Neurology, University of Minnesota, Minneapolis, Minnesota, USA; 6 Department of Psychiatry, University of Minnesota, Minneapolis, Minnesota, USA

**Keywords:** Mild Traumatic Brain Injury, Magnetoencephalography, Synchronous Neural Interactions, Mtbi Recovery, Individual Classification, Veterans

## Abstract

Mild traumatic brain injury (mTBI) affects 22% of U.S. service members returning from Afghanistan and Iraq. Its diagnosis is challenging due to the heterogeneous structural and functional alterations inflicted by diverse injury mechanisms. mTBI is diagnosed mainly based on history (trauma) and clinical evaluation, since conventional neuroimaging methods, such as magnetic resonance imaging (MRI) and computerized tomography (CT) of the brain, typically do not reveal clear abnormalities. Similarly, the assessment of recovery following mTBI relies exclusively on clinical evaluation, based on several criteria. With respect to brain function, we hypothesized that mTBI reflects disturbed dynamic interactions among neuronal populations, a disturbance not detectable by the aforementioned techniques. In a quest for an objective tool to detect the presence of mTBI and assess recovery from it, here we used magnetoencephalography (MEG), a modality highly suited to assess the dynamic functional status of the brain. Specifically, we used the Synchronous Neural Interactions (SNI) test to evaluate functional brain status of 257 healthy (“control”) veterans, 19 veterans with a clinical diagnosis of active mTBI (“a-mTBI”), and 18 veterans who suffered from mTBI and, at the time of testing, were deemed to have recovered from it (“r-mTBI”). A stepwise linear discriminant analysis (LDA) yielded 37 SNI predictors that classified 100% correctly of all 257 control and 19 a-mTBI brains. We then used these predictors to classify the 18 r-mTBI brains to control or a-mTBI groups: 9 brains (50%) were classified as control, whereas the other 10 (50%) were classified as a-mTBI. These findings (a) document the power of SNI MEG to correctly detect a-mTBI, and (b) raise concerns regarding the validity of clinical assessment tools to pronounce recovery from mTBI. On the positive side, our results provide an objective brain-based continuum along which the status of a mTBI brain can be assessed. This measure, together with clinical evaluation, should appreciably reduce the uncertainty and considerably improve the quantification of recovery from mTBI, guiding further treatment.

## Introduction

Since 2000, nearly 316,000 United States service members have experienced one or more mild traumatic brain injuries^1^, characterized by a range of physical, emotional, and cognitive symptoms. These symptoms typically persist for a few days to 3 months, but 47% of Afghanistan and Iraq service members reported that their symptoms persisted for more than 3 months.^2^

Currently, mTBI is incompletely understood and often difficult to diagnose. Heterogeneous injury mechanisms can produce diverse symptoms. Recent reviews have reported decidedly mixed results when attempting to detect structural or functional sequelae of mTBI using neuroimaging, primarily magnetic resonance imaging (MRI) and computerized tomography (CT).^3–4^ Uncertain diagnoses can impede clinical care. Frequently occurring comorbid physical and psychiatric disorders raise the challenge of differential diagnosis. Improved diagnostic techniques are clearly needed.

Magnetoencephalography (MEG) is an imaging technique with potential to improve diagnosis. When compared to other imaging approaches, MEG is noninvasive, and has high temporal (~1 ms) resolution.^5^ MEG can distinguish those with normal brain function from those with multiple brain-affecting disorders (e.g., post-traumatic stress disorder^6^) with high sensitivity and specificity. These brain-affecting disorders have unique “signatures”, characteristic alterations in cross-communication patterns among sensor pairs, or synchronous neural interactions (SNI).^7^ SNIs can be characterized globally or locally as representing hyper-correlated or decorrelated states between neuronal populations. A decorrelated network of neural interactions typifies a brain in a flexible state, able to process new information more readily. A hyper-correlated network reflects a more constricted brain state and one that is therefore less able to encode new information.

Several studies have sought MEG-based abnormalities associated with mTBI. Most report general decreases in neuronal network complexity,^8^ lack of cognitive reserve,^9^ or region-specific increases or decreases in functional connectivity.^10^ Dunkley, et al. (2015) proposed that MEG-identified network abnormalities may constitute a biomarker for mTBI.^11^ Because SNI patterns can distinguish groups with various brain affecting disorders from each other and from normal, we hypothesized that MEG could do the same with mTBI. We further hypothesized that MEG could assess, as an objective method, whether mTBI cases deemed “recovered” by clinical assessment were indeed grouped with controls, or, rather, with active mTBI.

## Materials and Methods

### Study participants

Male veterans participated in this study as paid volunteers (N = 294). The study protocol was approved by the relevant institutional review board and informed consent was obtained prior to the study. All TBI participants had completed a Comprehensive Traumatic Brain Injury Evaluation (CTBIE^12^) at the Minneapolis VAHCS. Exclusionary criteria included cardiac pacemakers or implanted ferrous metal, central nervous system disorders (e.g. Parkinson’s disease, cerebrovascular accidents, etc.), psychosis, or current alcohol or drug dependence. Those with a history of moderate to severe TBI, “behavioral health conditions” only, or “other conditions not related to behavioral health or TBI” were excluded. This yielded two mTBI groups: those currently experiencing mTBI symptoms (active mTBI, “a-mTBI”), and those who, on clinical assessment, were deemed to have recovered from previous mTBI (“r-mTBI”). Assignment to these two groups was based on providers’ endorsement of (a) “TBI with residual problems” or “combination of TBI and Behavioral Health conditions” (a-mTBI, N=19), or (b) “symptom resolution” (r-mTBI, N = 18). The a-mTBI group included 9 with PTSD and 3 with a depressive disorder; the r-mTBI group included 8 with concurrently diagnosed PTSD and 6 concurrently diagnosed with a depressive disorder. In the a-mTBI group, 42% were prescribed antidepressants, 42% sleep medications, and 26% pain medication (not including NSAIDs), with some participants prescribed a combination of such medications; in the r-mTBI group, 50% were prescribed antidepressants, 33% sleep medications, and 17% pain medications (not including NSAIDs), with some participants prescribed a combination of such medications. Controls were free from brain-affecting medical or mental health conditions. The interval (in months) between the participants’ most significant mTBI and the MEG scan was determined via chart review.

### Measures

#### Lifetime Trauma Exposure.

Lifetime trauma exposure was calculated from responses to the Deployment Risk and Resilience Inventory (DRRI).^13^ Specifically, 8 items from the Prior Stressors subscale (e.g., assault/sexual assault, prior combat, and natural disasters), 12 items from the Combat Experiences subscale assessing specific combat exposure, and 8 items from the Post Deployment Stressors subscale were summed to determine lifetime trauma exposure.

#### PCL.

PTSD symptoms were assessed using the PTSD Checklist – Civilian Version (PCL-C)^14^, a 17-item self-report scale assessing each PTSD symptom from the Diagnostic and Statistical Manual for Mental Disorders IV^15^. Participants rated how much they were bothered by each symptom in the past month using a 5-point Likert scale, ranging from “not at all” (1) to “extremely” (5). Item responses were summed to provide an index of current PTSD symptom severity.

#### BDI.

Depressive symptoms were assessed with the Beck Depression Inventory-Short Form (BDI-SF)^16^ a 13-item self-report questionnaire assessing the cognitive-affective aspects of depression. For each item, participants chose one of four response options indicating increasing symptom severity. Item scores range from 0 to 3 with a maximum total score of 39. The BDI-SF is one of the most widely used rating scales for depression.

#### MoCA.

The Montreal Cognitive Assessment (MoCA) assessed cognitive function. It is a screening instrument and tapping 8 different cognitive domains including attention and concentration, executive function, memory, language, visual / constructional skills, conceptual thinking, calculations, and orientation.^17^ Although all sections are brief, each contains items selected from longer psychometric instruments. The maximum possible score is 30 points. An extra point is added to the total score for patients with grade <12 education.

### Data acquisition

All participants underwent a MEG scan. As described previously,^7^ subjects lay supine within the electromagnetically shielded chamber and fixated their eyes on a spot ~ 65 cm in front of them, for 45–60s. MEG data were acquired using a 248-channel axial gradiometer system (Magnes 3600WH, 4-D Neuroimaging, San Diego, CA), band-filtered between 0.1 and 400 Hz, and sampled at 1017.25 Hz. Data with artifacts (e.g. from excessive subject motion) were eliminated from further analysis.

### Data analysis

Standard statistical methods were used to analyze the data, including analysis of covariance (ANCOVA) and linear discriminant analysis (LDA). The following packages were employed: IBM-SPSS statistical package, version 23^18^, Matlab (version R2015b)^19^, and ad hoc Fortran computer programs employing the International Mathematics and Statistics Library (IMSL; Rogue Wave Software, Louisville, CO, USA) statistical and mathematical libraries.

### MEG data processing

Processing of the raw MEG series was performed using programs in Python.^20^ Single trial MEG time series from all sensors underwent ‘prewhitening’^21^ using a (50,1,3) ARIMA model to obtain innovations (i.e. residuals).^20^ All possible pairwise zero-lag crosscorrelations (N = 30,628, given 248 sensors) were computed between the prewhitened MEG time series. Finally, the partial zero-lag crosscorrelations $PCC_{ij}^{0}$ (SNI) between i and j sensors were computed for all sensor pairs. $PCC_{ij}^{0}$ was transformed to $z_{ij}^{0}$ using Fisher’s^22^ z-transformation to normalize its distribution:

(1) $$\text{SNI}=z_{ij}^{0}=\text{atanh}\left(PCC_{ij}^{0}\right)$$

## ANCOVA

ANCOVA was used to evaluate SNI differences between the control and a-mTBI groups, where SNI was the dependent variable, Group was a fixed factor, and age was a covariate. For that purpose, SNIs were pooled from all subjects in each group.

## LDA

Of the three groups we studied, the control and a-mTBI groups were clinically well characterized; in contrast, r-mTBI comprised subjects who had undergone therapy for their mTBI and were now judged to be “normal”. In this analysis, we used the functional brain patterns (SNI test^7^) to assess the status of r-mTBI and assign them to the Control or a-mTBI group. For that purpose, we used the age-adjusted SNIs in a linear discriminant analysis, as follows. For each brain, there were 247 SNIs available for each one of the 248 sensors. For each sensor, we used the maximum and minimum SNI value ^23^ as input (N = 248 × 2 = 496 predictors) to a stepwise LDA to classify control and a-mTBI brains. This analysis yielded 100% correct classification of control and a-mTBI brains (see below). Hence, we used that discriminant function to classify the 18 r-mTBI brains. For each case (brain), we retained the probability of classification to a group and the ${\text{D}}^{2}$ Mahalanobis distances of each case from the center of the control and a-mTBI group centroids; the smaller of the two ${\text{D}}^{2}$ values indicates the group to which the case belongs (i.e. classified). Therefore, their ratio provides a measure of uncertainty of the classification: the lower the ratio $\frac{D^{2}smaller}{D^{2}larger}$, the more certain the classification assignment of the case. For quantitative comparisons, these ratios were log-transformed to normalize their distribution.

## Results

### General

The descriptive statistics of the 3 groups are given in Table 1. There were no statistically significant difference between the a-mTBI and r-mTBI groups (independent samples t-test) with respect to age (P = 0.294), PCL (P = 0.165), BDI (P = 0.091), and months from injury to scan (P = 0.255).

### Classification of control and a-mTBI brains

**Figure 1.**
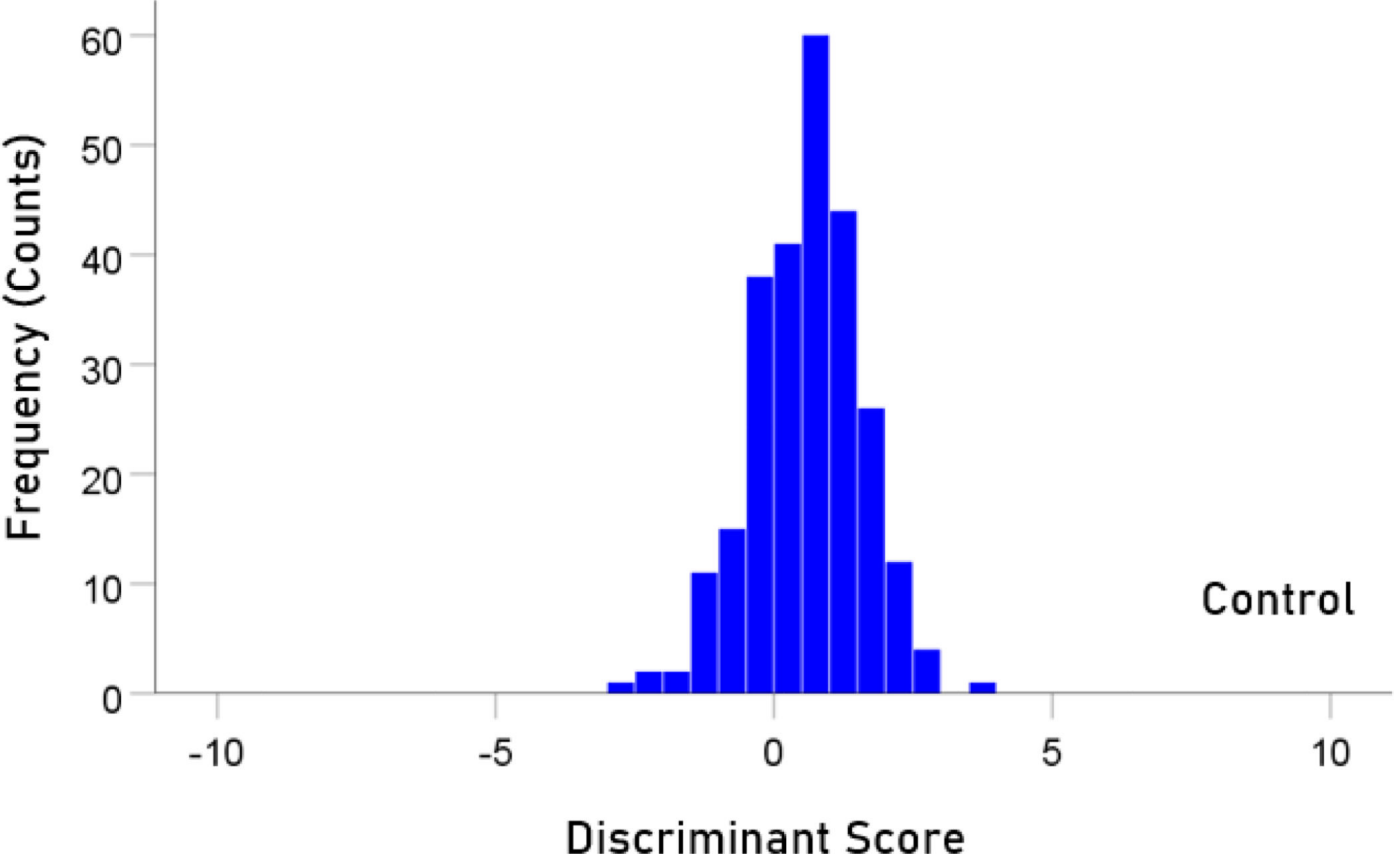
Frequency distribution of discriminant scores for the control group (N = 257).

**Figure 2.**
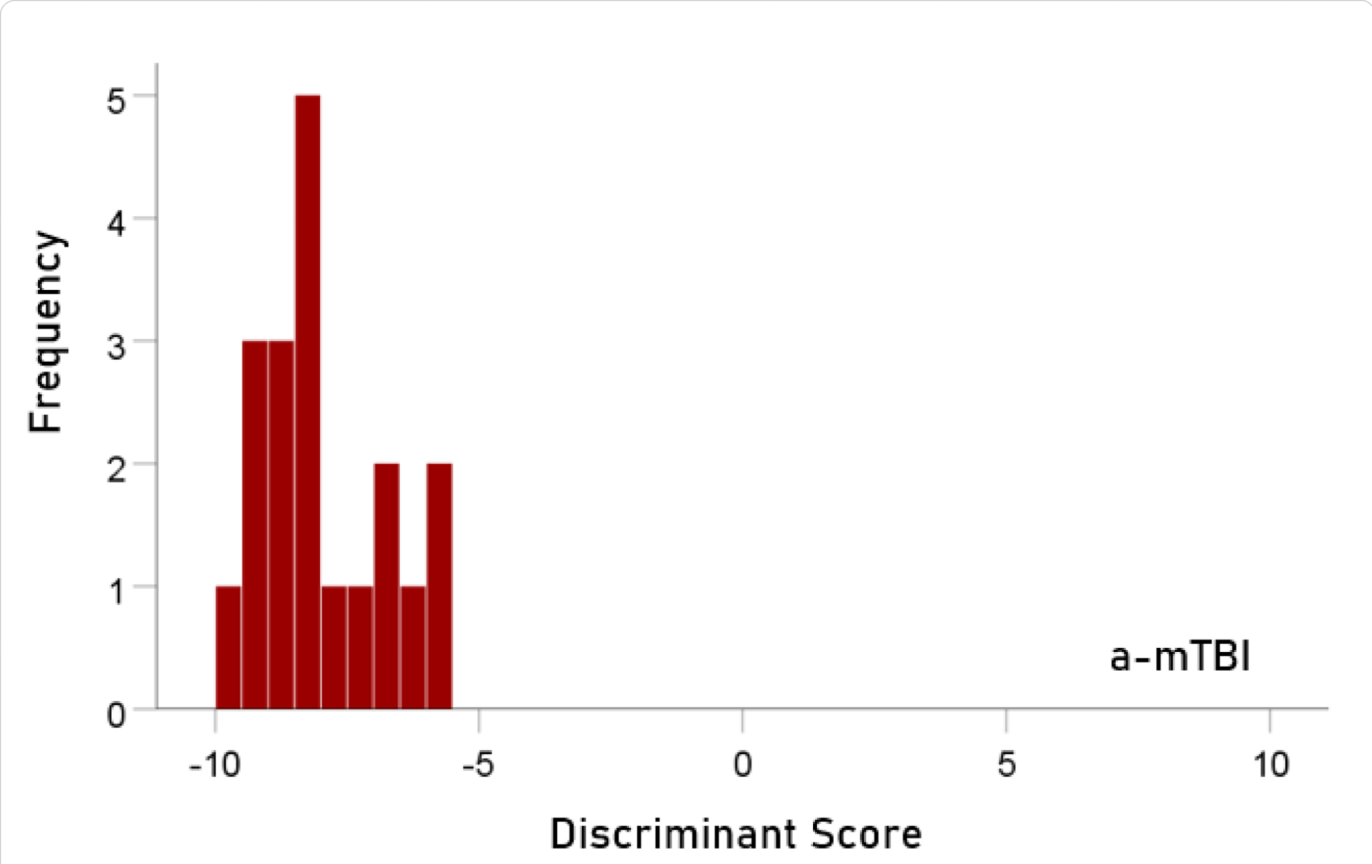
Frequency distribution of discriminant scores for the a-mTBI group (N = 19).

**Figure 3.**
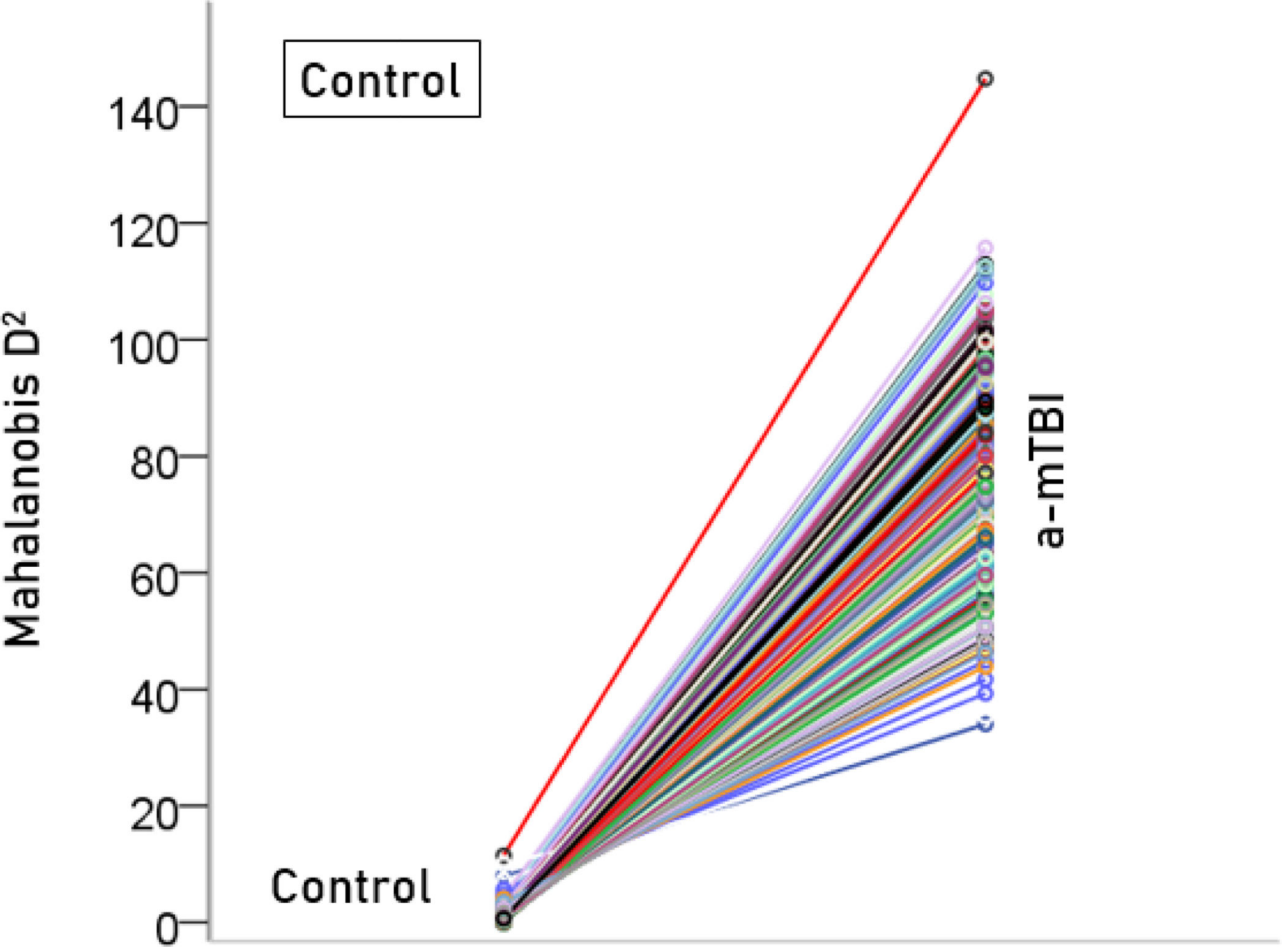
Mahalanobis ${\text{D}}^{2}$ values for the control group (N = 257).

**Figure 4.**
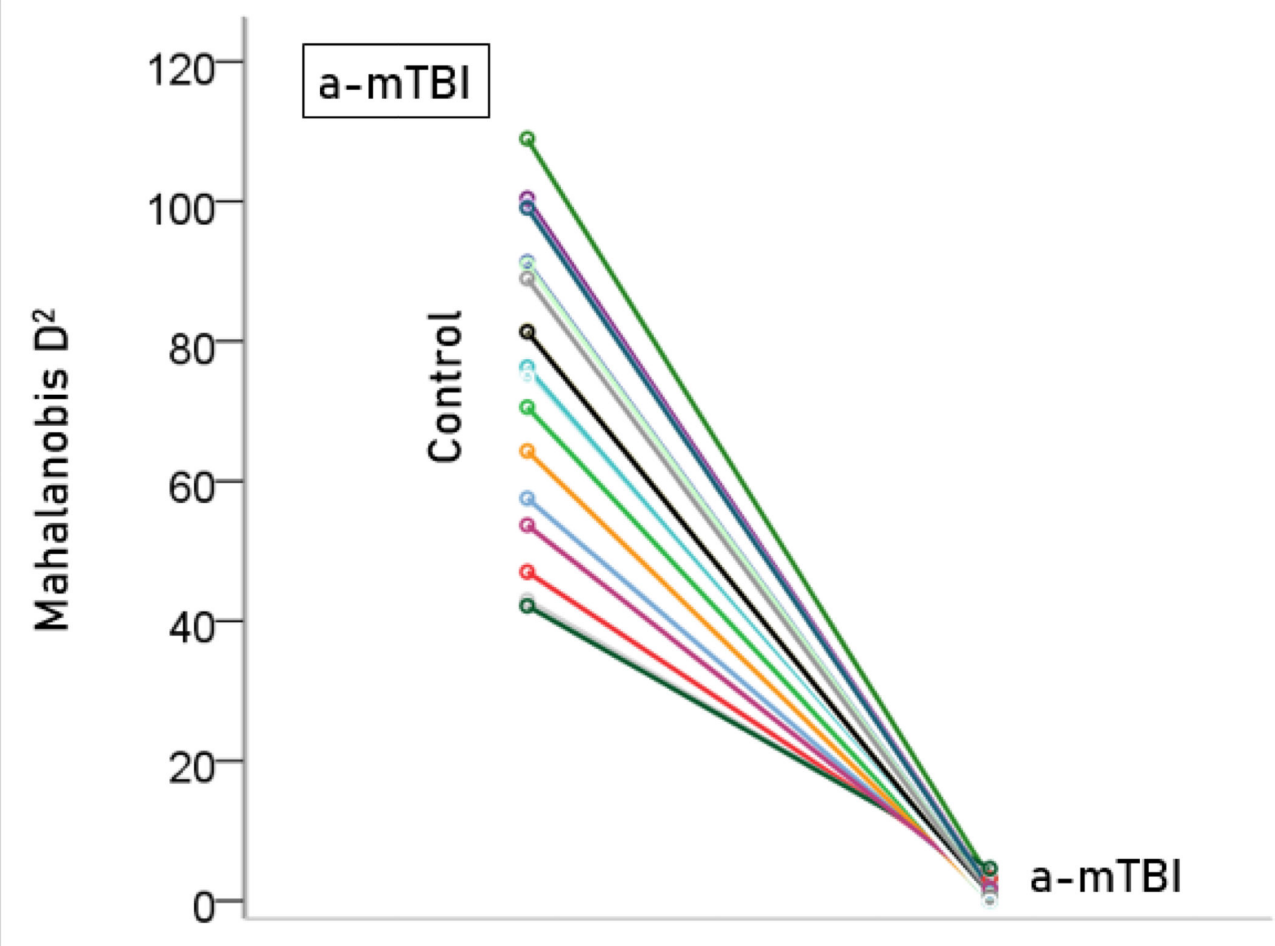
Mahalanobis ${\text{D}}^{2}$ values for the a-mTBI group (N = 19).

**Figure 5.**
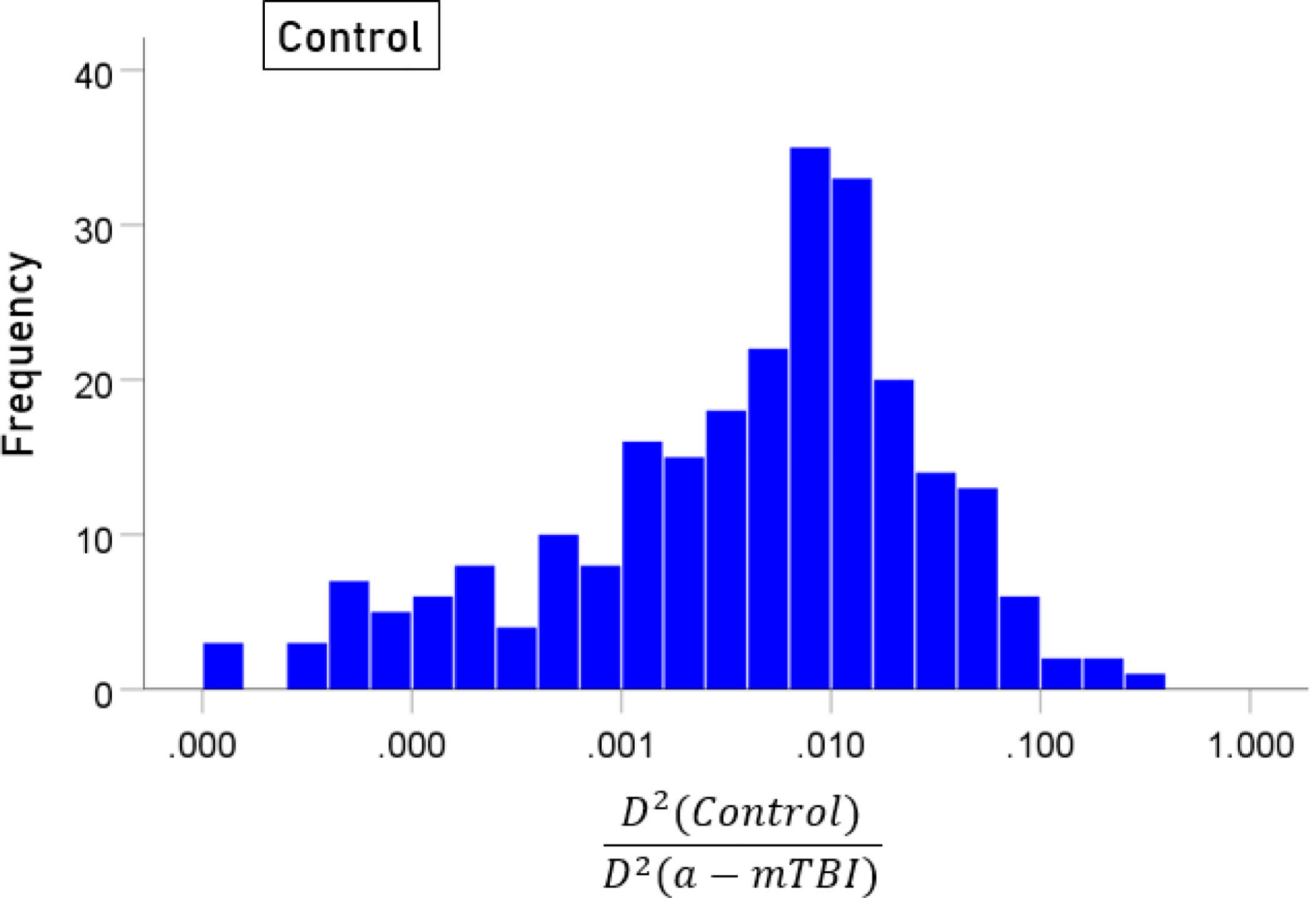
Frequency distribution of log-transformed $\frac{D^{2}smaller}{D^{2}larger}$ values for the control group (N = 257).

**Figure 6.**
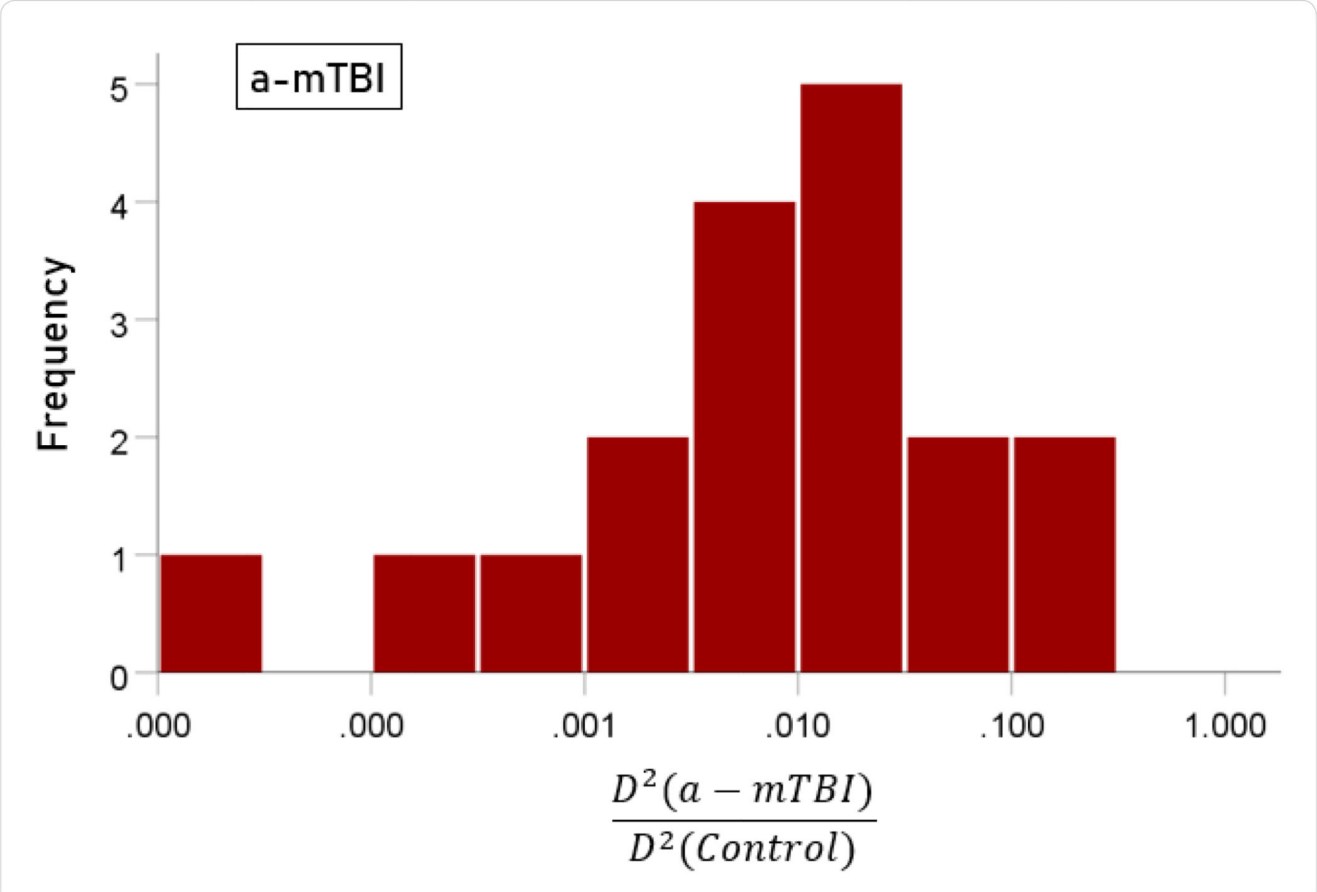
Frequency distribution of log-transformed $\frac{D^{2}smaller}{D^{2}larger}$ values for the a-mTBI group (N = 19).

The stepwise LDA yielded 100% correct classification of all 257 control and 19 a-mTBI brains with a probability of 1 for each brain, using 55/496 (11%) of the SNI predictors. A 100% correct classification was obtained in a cross-validation leave-one-out test. The frequency distribution of the discriminant scores for control and a-mTBI brains are shown in Figures. 1 and 2, respectively. It can be seen that they were tightly clustered and did not overlap. The ${\text{D}}^{2}$ Mahalanobis distances of the 257 control and 19 a-mTBI cases are shown in Figures. 3 and 4, respectively. The tight cluster of control and a-mTBI values, respectively, and the high values from the other group, attest to the high certainty of the classification outcome. Finally, the frequency distributions of the log-transformed $\frac{D^{2}smaller}{D^{2}larger}$ values for the control and a-mTBI classifications are shown in Figures. 5 and 6, respectively. The two distributions did not differ significantly (P = 0.278, independent samples t-test); this indicates that the classification performance was very similar for the two groups.

## Classification of r-mTBI brains

**Figure 7.**
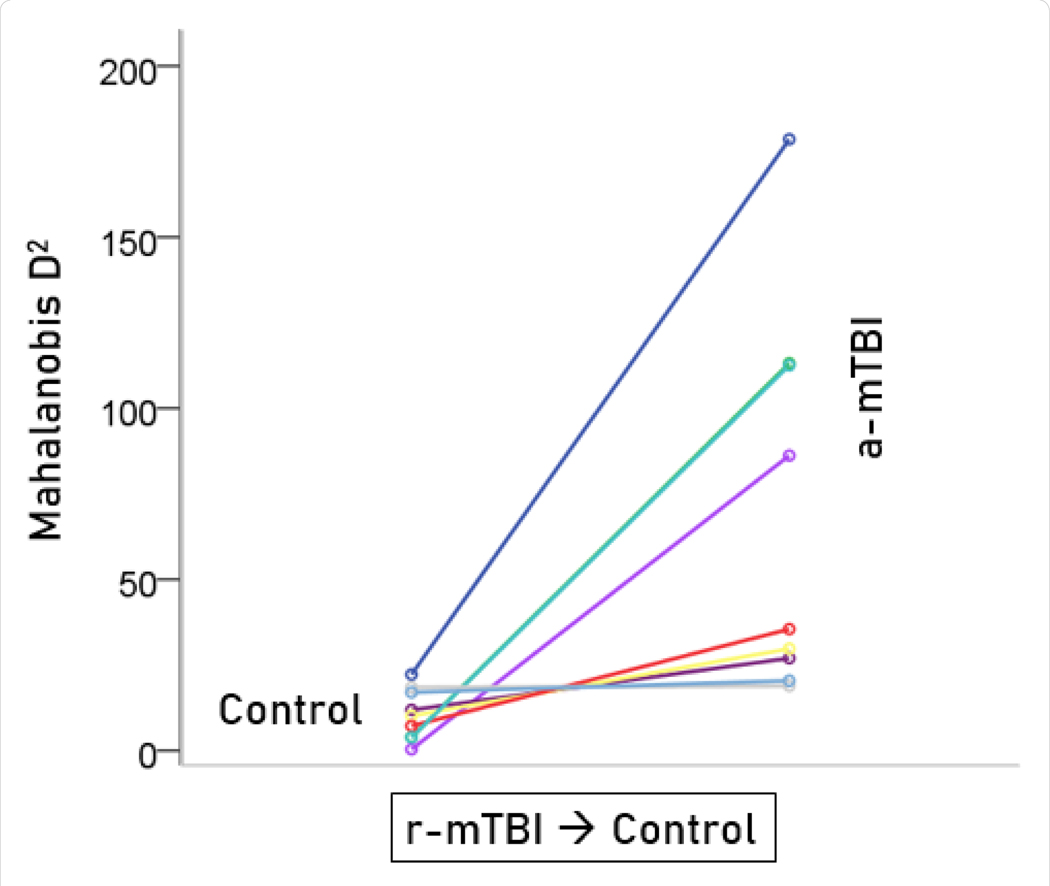
Mahalanobis ${\text{D}}^{2}$ values for the r-mTBI cases classified to the control group (N = 9).

**Figure 8.**
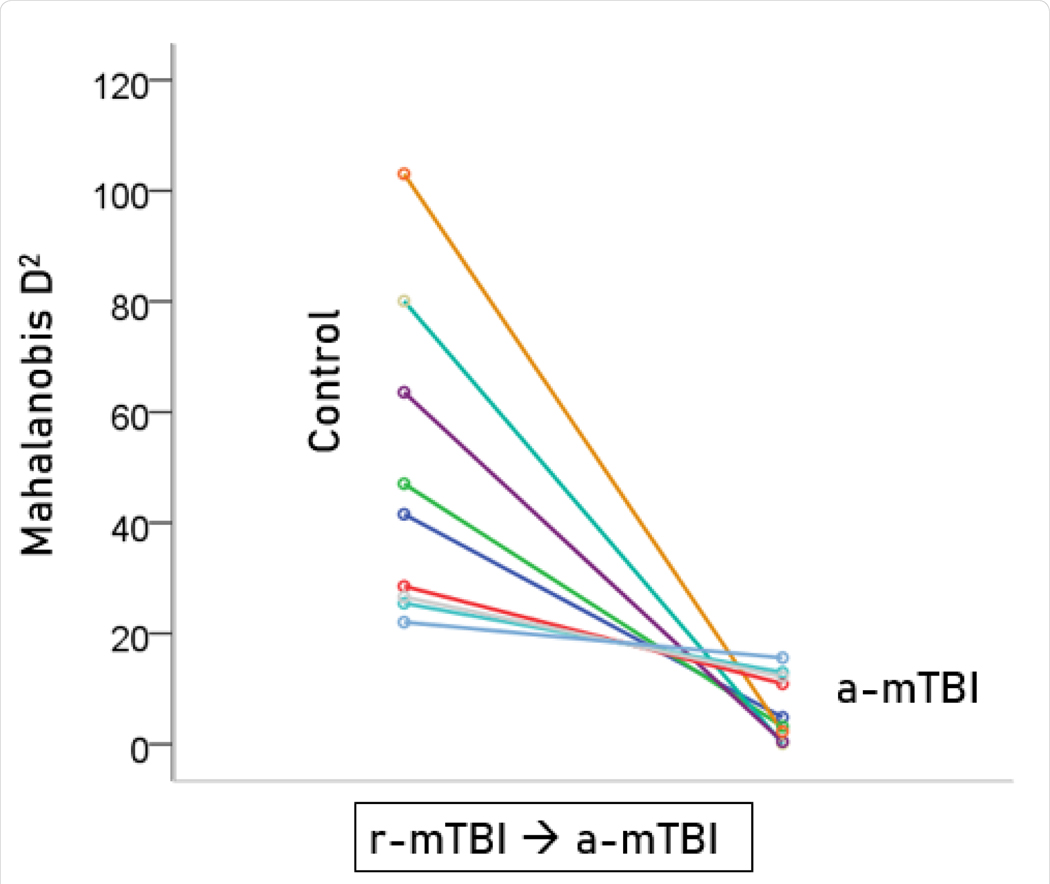
Mahalanobis ${\text{D}}^{2}$ values for the r-mTBI cases classified to the r-mTBI group (N = 9).

**Figure 9.**
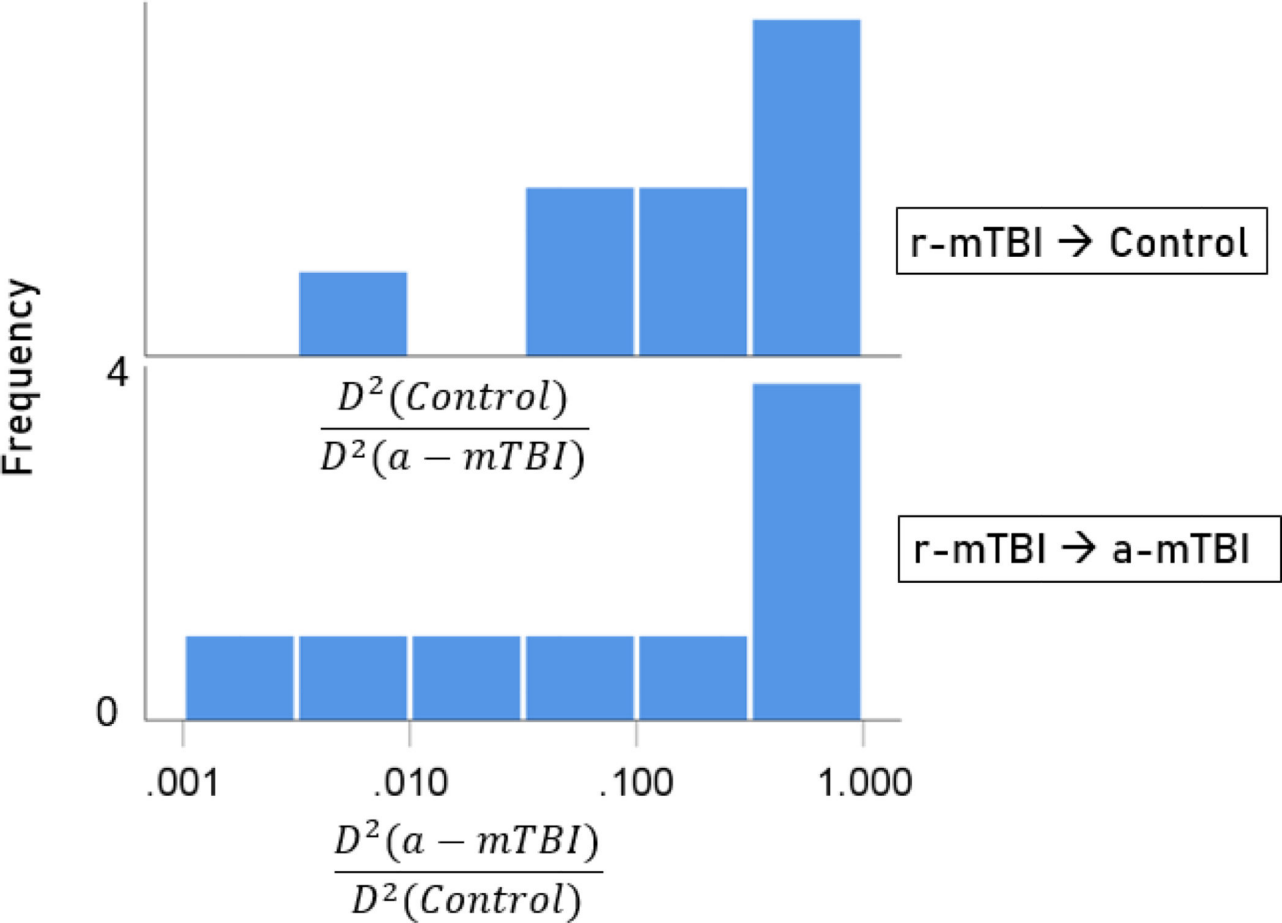
Frequency distribution of log-transformed $\frac{D^{2}smaller}{D^{2}larger}$ values for the 9 r-mTBI cases classified to the control group (top panel) and the 9 r-mTBI cases classified to the a-mTBI group (bottom panel).

Unlike control and a-mTBI brains, r-mTBI brains belong to a transitional category, along the a-mTBI → control continuum, undergoing therapy. A major objective of this study was to be able to classify those brains to control or a-mTBI groups and, in addition, assess the degree of certainty in this classification. Given that 55 unique SNIs provided 100% classification accuracy of the control and a-mTBI participants, we used the same SNIs to classify, we used it to classify each of the 18 r-mTBI cases. Indeed, 9 (50%) cases were classified as control and 9 (50%) as a-mTBI (Table 2). The Mahalanobis ${\text{D}}^{2}$ distances for the two classifications are shown in Figures. 7 and 8, respectively, and the frequency distributions of their log-transformed $\frac{D^{2}smaller}{D^{2}larger}$ values are shown in Figure 9. The two distributions did not differ significantly (P = 0.546, independent samples t-test). The 9 classified as a-mTBI complained of continued chronic headaches stemming from their mTBI, calling into question the accuracy of their “recovered” clinical diagnosis

## Discussion

There has been much debate over efforts to diagnose mTBI using neuroimaging. Eierud, et al recently concluded: “Despite the large efforts to date, neuroimaging methods still lack the individual patient-level sensitivity and specificity to serve as a diagnostic tool for mTBI”^4.^

Our findings may lend credence to its possible efficacy. Through functional neuroimaging and pairwise crosscorrelation, our results revealed a significant difference in SNIs between a currently impaired group of veterans with mTBI on the one hand, and a never-injured group on the other, demonstrating that MEG can make such a distinction at the group level. While other studies have identified comparable brain function alterations occurring soon after mTBI (6 days - 2 months post-injury),^24^ long-term alterations were observed in our a-mTBI group (7 months – 282 months post-injury).

In addition, the summative results of the LDA used in the individual classification yielded an overall effective analysis, but with a few misclassifications in the Recovered group. However, presence of continued chronic headaches stemming from their mTBI brought into question the accuracy of the initial “recovered” clinical diagnosis, therefore providing further confidence in the MEG analysis and classification. Based off these findings, MEG may be a useful diagnostic tool to aid clinician’s diagnostic decisions throughout the trajectory of an mTBI.

## Limitations and strengths

Our sample was composed of male veterans. Both mTBI groups were moderately small, had significant proportions affected by psychiatric comorbidity, brain-affecting medications, and long overall intervals between injuries and scans. Alternatively, this may be viewed as a strength in that it reflects the clinical realities of chronicity, medication, and comorbidity (PTSD and depression).

## Figures and Tables

**Table 1.** Descriptive statistics. (See text for abbreviations.)

Group	Age (y)	DRRI Total	PCL Total	BDI Total	MoCA Total	Injury to scan (months)
Control (No TBI) (N = 257) Mean SD	(257) 57.8 63.0 12.7	(208) 3.6 2.0 4.1	(245) 25.1 22.0 8.9	(246) 2.4 2.0 3.1	(213) 25.9 26.0 2.6	
r-mTBI (N = 18) Mean SD	(18) 36.6 11.8	(17) 9.8 4.0	(15) 57.2 13.6	(16) 13.6 5.9	(12) 26.2 2.8	110.6 134.0
a-mTBI (N = 19) Mean SD	(19) 41.4 15.1	(16) 8.6 5.3	(18) 48.8 19.1	(17) 9.8 6.6	(13) 26.2 2.1	69.8 72.8

**Table 2.** Classification statistics for the r-mTBI group. (See text for details.)

Participant	Group count	Classification	Probability
1	1	Control	1.000
2	2	Control	1.000
3	3	Control	1.000
4	4	Control	1.000
5	5	Control	1.000
6	6	Control	1.000
7	7	Control	0.999
8	8	Control	0.847
9	9	Control	0.550
10	1	a-mTBI	1.000
11	2	a-mTBI	1.000
12	3	a-mTBI	1.000
13	4	a-mTBI	1.000
14	5	a-mTBI	1.000
15	6	a-mTBI	1.000
16	7	a-mTBI	0.999
17	8	a-mTBI	0.998
18	9	a-mTBI	0.961

## References

[1] Defense and Veterans Brain Injury Center (DVBIC). DoD numbers for traumatic brain injury – worldwide totals. 2018. 2000–2018 Q1 as of June 21, 2018: https://dvbic.dcoe.mil/dod-worldwide-numbers-tbi (accessed 6-20-19).

[2] Schwab K, Terrio HP, Brenner LA, Epidemiology and prognosis of mild traumatic brain injury in returning soldiers. Neurology. 2017; 88: 1571–1579. 28314862 10.1212/WNL.0000000000003839

[3] Bigler ED, Finuf C, Abildskov TJ, Cortical thickness in pediatric mild traumatic brain injury including sports-related concussion. International Journal of Psychopathology. 2018; 132: 99–104. 10.1016/j.ijpsycho.2018.07.474 30040986

[4] Eierud C, Craddock RC, Fletcher S, Neuroimaging after mild traumatic brain injury: Review and meta-analysis. Neuroimage Clinical. 2014; 4: 283–294. 25061565 10.1016/j.nicl.2013.12.009 PMC4107372

[5] Leahy RM, Mosher JC, Spencer ME, A study of dipole localization accuracy for MEG and EEG using a human skull phantom. Electroencephalography & Clinical Neurophysiology. 1998; 107: 159–173. 9751287 10.1016/s0013-4694(98)00057-1

[6] Engdahl B, Leuthold AC, Tan HM, Post-traumatic stress disorder: A right temporal lobe syndrome? Journal of Neural Engineering. 2010; 7(6): 1–8. 10.1088/1741-2560/7/6/066005 20980718

[7] Georgopoulos AP, Karageorgiou E, Leuthold AC, Synchronous neural interactions assessed by magnetoencephalography: A functional biomarker for brain disorders. Journal of Neural Engineering. 2007; 4: 349–355. 18057502 10.1088/1741-2560/4/4/001

[8] Luo Q, Xu D, Roskos T, Complexity analysis of resting state magnetoencephalography activity in traumatic brain injury patients. Journal of Neurotrauma. 2013; 30: 1702–1709. 23692211 10.1089/neu.2012.2679 PMC3796321

[9] Pang EW, Dunkley BT, Doesburg SM, Reduced brain connectivity and mental flexibility in mild traumatic brain injury. Annals of Clinical and Translational Neurology. 2016; 3(2): 124–131. 26900581 10.1002/acn3.280 PMC4748313

[10] Huang M, Harrington DL, Swan AR, Resting-state magnetoencephalography reveals different patterns of aberrant functional connectivity in combat-related mild traumatic brain injury. Journal of Neurotrauma. 2017; 34: 1412–1426. 27762653 10.1089/neu.2016.4581

[11] Dunkley BT, Costa LD, Bethune A, Low-frequency connectivity is associated with mild traumatic brain injury. Neuroimage Clinical. 2015; 3(2): 124–31. 10.1016/j.nicl.2015.02.020 PMC4379387 25844315

[12] DePalma RG, Hoffman SW. Combat blast related traumatic brain injury (TBI): Decade of recognition; promise of progress. Behavioral Brain Research. 2018; 340: 102–105. 10.1016/j.bbr.2016.08.036 27555540

[13] King LA, King DW, Vogt DS, Deployment Risk and Resilience Inventory: A collection of measures for studying deployment-related experiences of military personnel and veterans. Military Psychology. 2006; 18(2): 89–120.

[14] Weathers FW, Litz B, Huska JA, The PTSD Checklist—Civilian Version (PCL-C) for DSM-IV. Boston, Behavioral Sciences Division, National Center for PTSD. 1991.

[15] American Psychiatric Association: Diagnostic and Statistical Manual of Mental Disorders: DSM-IV-TR. Washington, DC, 2000.

[16] Beck AT, Beck RW: Screening depressed patients in family practice. A rapid technique. Postgraduate Medicine. 1972; 52: 81–5. 4635613 10.1080/00325481.1972.11713319

[17] Nasreddine ZS, Phillips NA, Bedirian V, The Montreal Cognitive Assessment, MoCA: A brief screening tool for mild cognitive impairment. Journal of the American Geriatric Society. 2005; 53: 695–699. 10.1111/j.1532-5415.2005.53221.x 15817019

[18] IBM Corp. Released 2015. IBM SPSS Statistics for Windows, Version 23.0. Armonk, NY: IBM Corp.

[19] MATLAB and Statistics Toolbox Release 2015b, The MathWorks, Inc., Natick, Massachusetts, United States.

[20] Mahan MY, Leuthold AC, Georgopoulos AP. Spatiotemporal brain network analysis of healthy humans based on magnetoencephalography and functional MRI in the resting state. BMC Neuroscience. 2015; 16(Suppl 1): 155.

[21] Box GEP, Jenkins GM. Time Series Analysis: Forecasting and Control. San Francisco, CA: Holden-Day; 1976.

[22] Fisher RA. Statistical Methods for Research Workers. 13th ed. Edinburgh, Scotland: Oliver & Boyd; 1958.

[23] Engdahl B, James L, Miller R, A Magnetoencephalographic (Magnetoencephalography) Study of Gulf War Illness. EBioMedicine. 2016; 12: 127–132. 27592598 10.1016/j.ebiom.2016.08.030 PMC5078573

[24] Kaltiainen H, Helle L. Theta-band oscillations as an indicator of mild traumatic brain injury. Brain Topography. 2018; 31: 1037–1046. 30097835 10.1007/s10548-018-0667-2 PMC6182433

[25] Christova P, James LM, Engdahl B, Diagnosis of posttraumatic stress disorder (PTSD) based on correlation of prewhitened fMRI data: Outcomes and areas involved. Experimental Brain Research. 2015; 233: 2695–2705. 26070898 10.1007/s00221-015-4339-0

